# UHRF1-mediated ubiquitination of nonhomologous end joining factor XLF promotes DNA repair in human tumor cells

**DOI:** 10.1016/j.jbc.2024.107823

**Published:** 2024-09-27

**Authors:** Zhiwen Deng, Caiyun Long, Shuzhen Han, Zhishen Xu, Teng Hou, Weili Li, Xingwu Wang, Xiangyu Liu

**Affiliations:** 1 International Cancer Center, Guangdong Key Laboratory of Genome Instability and Human Disease Prevention, Marshall Laboratory of Biomedical Engineering, Department of Biochemistry and Molecular Biology, Shenzhen University Medical School, Shenzhen, China; 2 South China Hospital, Health Science Center, Shenzhen University, Shenzhen, China; 3 Molecular Cancer Research Center, School of Medicine, Sun Yat-Sen University, Shenzhen, Guangdong, China; 4 Department of Hematology, The Second People's Hospital of Shenzhen, Shenzhen, China

**Keywords:** UHRF1, XLF, ubiquitination, DNA repair, K63

## Abstract

UHRF1 (Ubiquitin-like with PHD and Ring Finger domains 1) is a crucial E3 ubiquitin ligase and epigenetic regulator with pivotal roles in various biological processes, including the maintenance of DNA methylation, regulation of gene expression, and facilitation of DNA damage repair. In this study, we unveil that UHRF1 interacts with the nonhomologous end joining factor XLF (also known as Cernunnos) following DNA double strand breaks in HeLa cells. Furthermore, we demonstrate that UHRF1 catalyzes lysine 63-linked polyubiquitination of XLF, rather than lysine 48-linked polyubiquitination. Notably, this polyubiquitination of XLF by UHRF1 does not affect its protein stability; instead, it enhances the recruitment of XLF to the sites of DNA damage. These findings shed light on the role of UHRF1 as a novel regulator of DNA repair through XLF in tumor cells.

Posttranslational modifications including ubiquitination are extremely important for DNA damage repair (1, 2). For example, FBXW7 E3 ligase promoted K63 polyubiquitylation of XRCC4 at lysine(K) 296 and enhanced its association with the Ku70/80 complex to facilitate nonhomologous end joining (NHEJ) repair (3). UHRF1 is an E3 ubiquitin ligase that consists of multiple domains including N-terminal ubiquitin-like domain, tandem Tudor domain, the plant homodomain, the Set and Ring-Associated domain, and the C-terminal RING domain that harbors intrinsic E3 ligase activity toward several downstream targets including histones and nonhistone substrates (4, 5, 6).

UHRF1 plays an essential role in epigenetic regulation such as DNA methylation maintenance, by recruiting DNA methyltransferase DNMT1 to hemimethylated DNA and H3K9me2/3 (7). Also, UHRF1 interacts with several epigenetic factors including G9a, SUV39H1, HDAC1, USP7/HAUSP, PARP1, and TIP60, suggesting its essential roles in epigenetic functions and regulations (6, 8, 9, 10, 11, 12). In physiological conditions, the protein levels of UHRF1 are regulated during cell cycle. UHRF1 expression peaks in the S phase while DNA replication occurs and is downregulated at the end of the M phase through S652 phosphorylation (9). UHRF1 is present only in actively proliferating tissues and kept in an undetectable level in terminally differentiated tissues (13, 14). However, in several types of cancers, UHRF1 is overexpressed throughout all the cell cycle phases and executes a great impact on tumorigenesis and cancer progression (15, 16), with unknown mechanisms.

DNA repair machineries are crucial for maintaining the integrity of the genome and preventing genetic damage (17). Several studies showed that UHRF1 was involved in DNA repair through multiple pathways. Depletion of UHRF1 in cells resulted in hypersensitivity to DNA damage and increased occurrence of chromosomal aberrations as well as sister chromatid exchange, indicating its role for genome stability (18, 19). UHRF1 has also been shown to influence the function of DNA repair–related proteins, such as p53, which are critical for cell cycle regulation and tumor suppression (20). Zhang *et al.* reported that UHRF1 functions downstream of BRCA1 and is important for BRCA1-mediated removal of RIF1 from double strand breaks (DSBs) to promote homologous recombination (21).

XLF is a “core” NHEJ factor that is essential for end-ligation and conserved in all eukaryotes (22). During NHEJ, XLF plays critically complementary functions with other DNA repair factors such as ATM, 53BP1, and PAXX (Paralog of XRCC4 and XLF) (23, 24, 25). For example, Xlf^−/−^Paxx^−/−^ mice displayed severe genomic instability and embryonic lethality (25). Importantly, XLF is sophisticatedly regulated by posttranslational modifications. For example, XLF was phosphorylated by Akt at T181 and dissociated from XRCC4/Lig4 complex and was translocated to cytoplasm, impeding NHEJ efficiency (26). In the present study, we demonstrated that the UHRF1 directly interacted with XLF and mediated K63-linked polyubiquitination. The ubiquitination of XLF was critical for its recruitment at sites of DNA damage.

## Results

### UHRF1 is recruited to chromatin upon DNA damage in cancer cells

**Figure 1:**
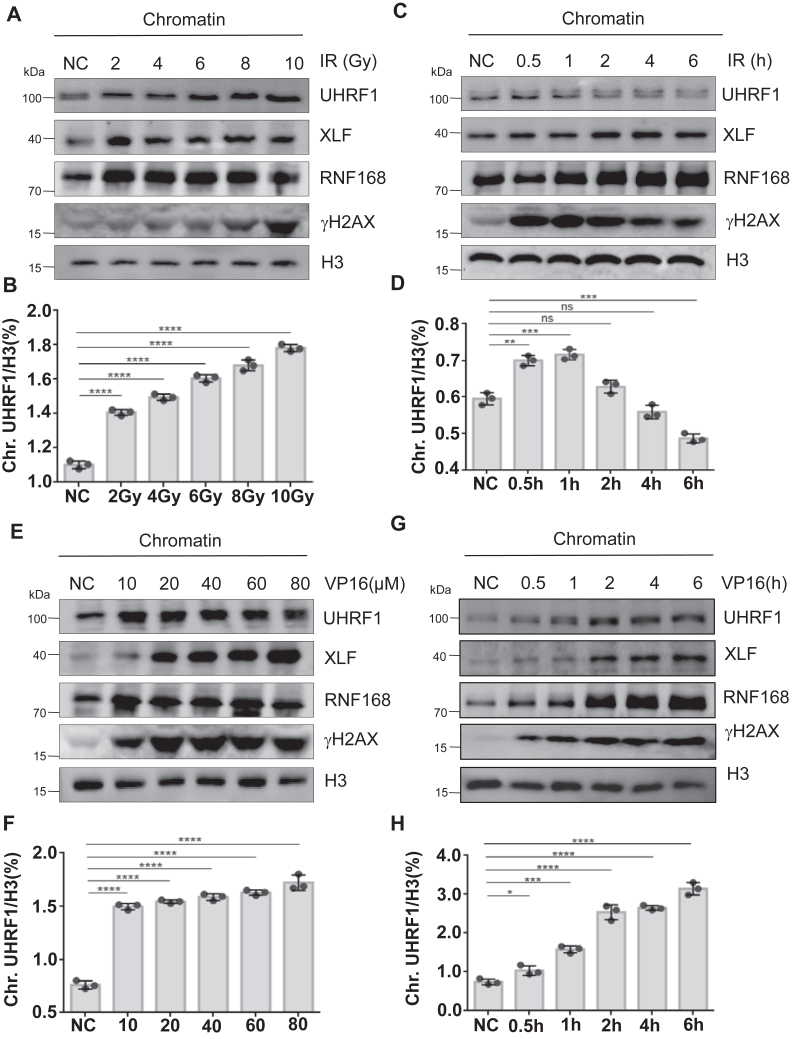
**UHRF1 is recruited to chromatin upon DNA damage in cancer cells**. *A* and *B*, HeLa cells were exposed to increasing doses of ionizing radiation (IR), and chromatin was extracted 30 min post-release for Western Blot. *B*, statistical analysis of the signal intensities from three independent experiments shown in (*A*) (*t* test). ∗∗∗∗*p* < 0.0001. *C* and *D*, HeLa cells were exposed to 10 Gy IR, and at specified time points, cells were harvested, chromatin was extracted, and Western Blot was performed. *D*, statistical analysis of the signal intensities from three independent experiments shown in (*C*) (*t* test). ∗∗*p* < 0.01; ∗∗∗*p* < 0.001. *E* and *F*, HeLa cells were treated with a specified concentration of VP16 for 2 h, and chromatin was extracted for Western Blot. *F*, statistical analysis of the signal intensities from three independent experiments shown in (*C*) (*t* test). ∗∗∗∗*p* < 0.0001. *G* and *H*, HeLa cells were treated with 40 μM VP16, and at specified time points, cells were harvested, chromatin was extracted, and Western Blot was performed. *H*, statistical analysis of the signal intensities from three independent experiments shown in (*C*) (*t* test). ∗*p* < 0.05; ∗∗∗*p* < 0.001; ∗∗∗∗*p* < 0.0001.

In response to DNA damage, the composition of chromatin was usually changed to facilitate the access of DNA repair factors for the repair progress (27). To test whether UHRF1 participate in DSBs, we treated human cervical cancer HeLa cells with increasing doses of irradiation (IR). We found that 2 to 10 Gy of irradiation sufficiently induced phosphorylation of H2AX at Ser 139 (γH2AX) at chromatin, a marker of DNA damage, 30 min after treatment. Interestingly, we found that UHRF1 was increasingly recruited to the chromatin after IR treatment. RNF168, a novel chromatin-associated E3 ligase, also assembled at DSBs as previously reported (28). As an essential NHEJ core factor, XLF was also found to be accumulated at chromatin after IR treatment (Fig. 1, *A* and *B*). Next, we treated the cells with 10 Gy of irradiation and incubated at tissue culture conditions for a certain time as γH2AX levels were decreased after 4 to 6 h of incubation. We found that UHRF1 was recruited to the chromatin 0.5 to 4 h after irradiation and left the chromatin after 6 h, suggesting the dynamic recruitment and turnover of UHRF1 around the DSBs sites (Fig. 1, *C* and *D*). We also treated the cells with topoisomerase II inhibitor etoposide (VP16) to generate DSBs and found that both UHRF1 and XLF were recruited to chromatin in dose- and time-dependent manners after VP16 treatment (Fig. 1, *E*–*H*). Finally, we extracted whole cell lysate from the previous samples and found that total protein levels of UHRF1 and XLF were not changed (Fig. S1, *A* and *B*), excluding the possibility that DNA damage affected the stability of UHRF1 protein levels. Together, these results indicated that UHRF1 participated in DNA damage response and could play essential roles in DNA repair.

### UHRF1 interacts with XLF and is important for DNA damage repair

**Figure 2:**
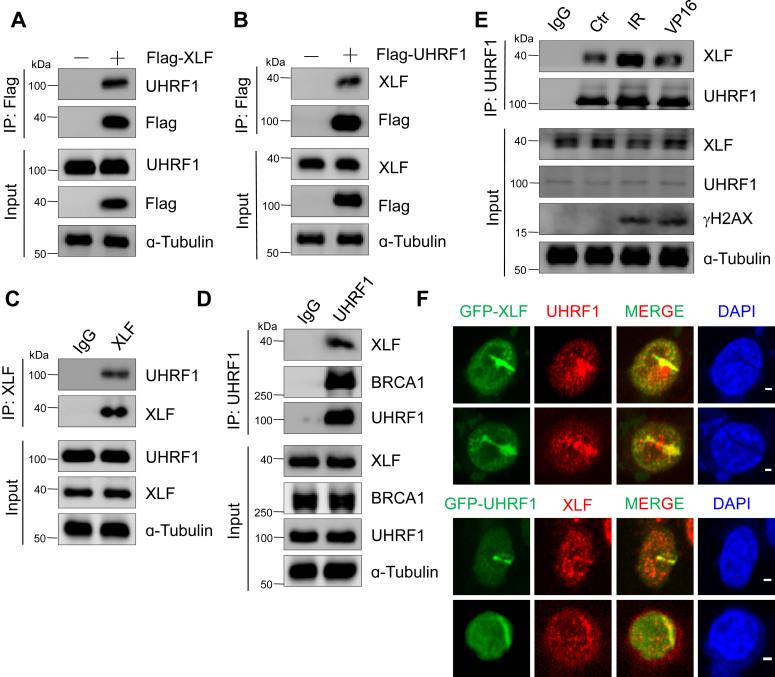
**UHRF1 interacts with XLF and is important for DNA damage repair**. *A* and *B*, after overexpressing Flag-XLF or Flag-UHRF1 in HeLa cells for 48 h, whole-cell lysates were extracted by adding BenzoNuclease to the cell lysate. Subsequently, immunoprecipitation (IP) was performed with anti-Flag antibody, followed by Western Blot. *C* and *D*, whole-cell lysate was extracted from HeLa cells, and immunoprecipitation was performed with anti-XLF antibody or anti-UHRF1 antibody, followed by Western Blot using the indicated antibody. *E*, HeLa cells, either untreated or exposed to 10 Gy IR for 30 min, followed by treatment with 80 μM VP16 for 2 h, had whole-cell lysates extracted. Immunoprecipitation was carried out with anti-UHRF1 antibody, followed by Western Blot using the indicated antibody. *F*, after overexpressing GFP-XLF (*up*) or GFP-UHRF1 (*down*) in HeLa cells for 24 h, immunofluorescence analysis (IF) was performed with anti-UHRF1 antibody or anti-XLF antibody, Scale bars represent 10 μm.

Previous reports showed that UHRF1 interacted with BRCA1 that participated in homologous recombination repair (21). To fully understand the function of UHRF1 in DNA damage repair, we expressed Flag-labeled UHRF1 or XLF vectors in HeLa cells and performed immunoprecipitation (IP) experiments after DNA damage. Surprisingly, we found that Flag-UHRF1 pulled down endogenous XLF and *vice versa* (Fig. 2, *A* and *B*). Endogenous IP experiment also proved the interaction between UHRF1 and XLF (Fig. 2, *C* and *D*). Notably, interaction between UHRF1 and XLF was enhanced in response to DNA damage (Fig. 2*E*). In addition, we individually transfected HeLa cells with GFP-labeled UHRF1 or XLF and performed the laser micro-irradiation experiment. After generating DNA damage, we immediately fixed the cells and then immuno-stained the cells. We found that both exo- and endo-expressed UHRF1 and XLF colocalized at the laser stripes (Fig. 2*F*). These results suggested that UHRF1 interacted with XLF *in vivo*. Importantly, the interaction was enhanced after DNA damage, implying that these two proteins might coordinate to play important roles during DNA repair.

### UHRF1 promotes XLF ubiquitination *via* K63 linkage

**Figure 3:**
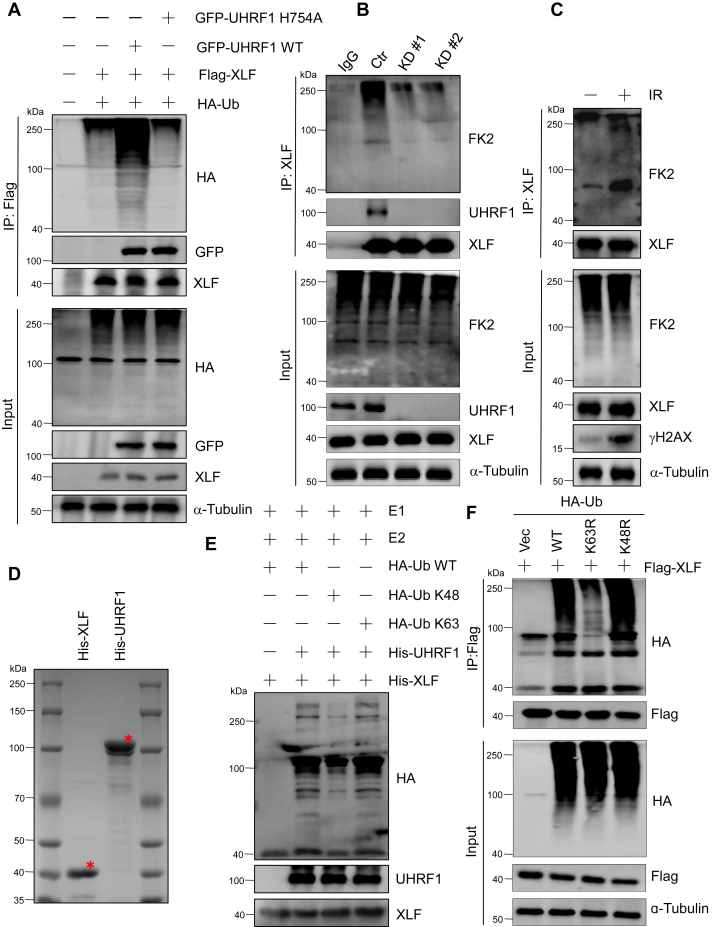
**UHRF1 promotes XLF ubiquitination *via* K63 linkage**. *A*, after transfecting HeLa cells with the above-mentioned plasmid for 48 h, the cells were exposed to 10 Gy of IR and then released for 30 min. Immunoprecipitation was performed under denaturing conditions, followed by Western Blot analysis to detect changes in XLF ubiquitination levels. *B*, after knocking down UHRF1 in HeLa cells, the level of XLF ubiquitination was detected. Before extracting whole-cell lysates, the cells were exposed to 10 Gy IR and allowed to recover for 30 min. *C*, whole-cell lysate was extracted from HeLa cells, either untreated or exposed to 10 Gy IR and released for 30 min. Immunoprecipitation was performed using anti-XLF antibody, and the ubiquitination levels of XLF were detected by Western Blot. *D*, we expressed His-tagged vectors in *Escherichia coli* and performed Coomassie blue staining after protein purification (asterisk indicated purified protein). *E*, the ubiquitination of XLF by UHRF1 *in vitro* was performed in a reaction system containing E1, E2, and ubiquitin (See Experimental procedures). *F*, following the overexpression of Flag-XLF and different HA-ubiquitin constructs in HeLa cells for 48 h, cells were exposed to 10 Gy IR, released for 30 min, and whole-cell lysates were extracted for anti-Flag immunoprecipitation. Changes in XLF ubiquitination levels were examined by Western Blot.

Considering that UHRF1 is an E3 ubiquitin ligase (29) as well as that UHRF1 interacts with XLF, we consider whether XLF could be ubiquitinated by UHRF1. To test this hypothesis, we cotransfected HeLa cells with Flag-XLF, WT UHRF1, and E3 enzymatic dead mutant UHRF1-H754A, together with exogenous expressed HA-Ubiquitin. By performing protein immunoprecipitation under denaturing conditions, we found that the expression of WT UHRF1 increased the ubiquitination level of XLF, while UHRF1-H754A did not affect the ubiquitination level of XLF (Fig. 3*A*). It is also true for protein immunoprecipitation under normal conditions (Fig. S2*A*), suggesting that UHRF 1 promotes the ubiquitination of XLF. Then we knocked down UHRF1 by CRISPR techniques using two independent pairs of guided-RNA and immunoprecipitated XLF for the detection of ubiquitination using FK2 antibodies. We found that depletion of UHRF1 sufficiently reduced ubiquitination levels of XLF (Fig. 3*B*). In addition, after we treated the cells with 10 Gy of irradiation, we found that ubiquitination of XLF was enhanced after DNA damage (Fig. 3*C*). To verify whether UHRF1 ubiquitinates XLF directly, we first purified His-tagged XLF and UHRF1 proteins (Fig. 3*D*) and then performed *in vitro* ubiquitination assays (Fig. 3*E*). The results demonstrated that UHRF1 directly mediated the ubiquitination of XLF. Next, we conducted ubiquitination experiments using ubiquitin mutant vectors, such as K48R or K63R mutants where the lysine residues at position 48 or 63 of ubiquitin were mutated to arginine, leaving other lysine residues unchanged. Alternatively, we utilized ubiquitin multipoint mutants at K48 or K63, where only the lysine residue at position 48 or 63 of ubiquitin was preserved while all other lysine residues were mutated to arginine to simulate deubiquitination. We found that XLF ubiquitination was K63-linked rather than K48-linked, which was also confirmed by cell-based experiments (Figs. 3*F* and S2*B*). Considering that XLF protein levels were not changed after co-expression with UHRF1 (Figs. 3*A* and S2*A*), depletion of UHRF1(Fig. 3*B*), or DNA damage (Fig. 3*C*), we suspected that ubiquitination may not result in the degradation of the XLF protein but rather is likely to play other roles in regulating XLF functions.

### XLF ubiquitination does not lead to its degradation

**Figure 4:**
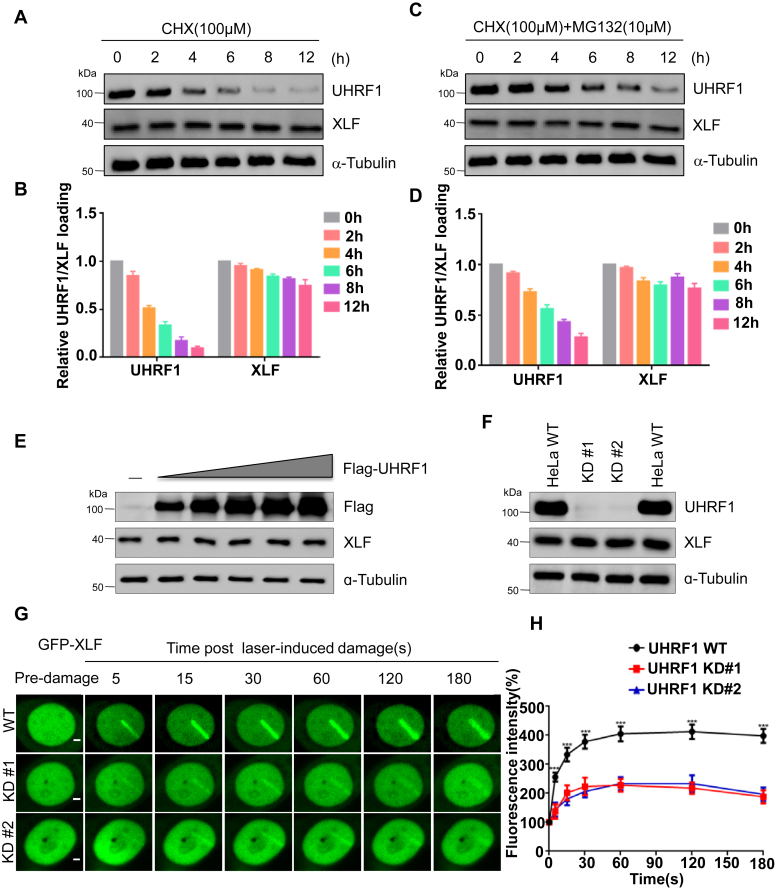
**XLF ubiquitination does not lead to its degradation**. *A* and *B*, HEK293T cells were treated with cycloheximide (CHX), and cells were harvested at specified time points to extract whole-cell lysates. Changes in the levels of UHRF1 and XLF proteins were detected through Western Blot. *B*, statistics of the signal intensities for the experiment in (*A*). *C* and *D*, HEK293T cells were treated with CHX and MG132, and cells were harvested at specified time points to extract whole-cell lysates. Changes in the levels of UHRF1 and XLF proteins were assessed by Western Blot. *D*, statistics of the signal intensities for the experiment in (C). *E*, gradient overexpression of Flag-UHRF1 in HeLa cells for 48h, followed by the extraction of whole-cell lysates and assessment of changes in XLF protein levels through Western Blot. *F*, UHRF1 was knocked down in HeLa cells using CRISPR-Cas 9 technology, and UHRF1 knockdown efficiency and XLF protein levels were determined by Western Blot. *G* and *H*, HeLa WT cells and UHRF1 knock down cells were transfected with GFP-XLF. After 24 h, dynamic recruitment of XLF to DNA damage tracks were monitored using laser micro-irradiation–coupled live-cell imaging, Scale bars represent 10 μm. *H*, we analyzed and quantified the percentage of fluorescence increase along 20 cellular DNA damage trajectories.

To confirm the roles of XLF ubiquitination, we treated the cells with cycloheximide at different time courses to block protein synthesis, we did not see a significant degradation of XLF, suggesting a relatively long half life of XLF. In contrast, UHRF1 levels decreased after 4 h treatment of cycloheximide, consistent with the previous report (Fig. 4, *A* and *B*) (30, 31). Interestingly, degradation of UHRF1 was mediated by proteasome degradation pathway, since the protein levels of UHRF1 were rescued by adding the proteasome inhibitor MG132 (Fig. 4, *C* and *D*), but not by adding the lysosome inhibitor chloroquine (Fig. S3, *A* and *B*). To further confirm the role of UHRF1 on XLF, we expressed increasing amount of UHRF1 and did not see change of XLF expression (Fig. 4*E*). At the same time, knock down of UHRF1 did not decrease XLF levels (Fig. 4*F*). In contrast to K48-linked polyubiquitin chains, which usually led to proteasome-directed protein degradation, K63-linked ubiquitin chains were more implicated in the DNA damage response for signal transduction. To investigate the role of UHRF1 on XLF, we expressed GFP-labeled XLF into WT or UHRF1 KD HeLa cells and performed the laser micro-irradiation experiment. We found that while GFP-XLF was robustly recruited to the DSBs site shortly (5s) after exposure of laser in WT cells, in two independent UHRF1 KD cell lines, recruitment of XLF to DSBs was greatly decreased. We collected at least twenty cells and the results showed significant differences (Fig. 4, *G* and *H*).

### UHRF1 promotes XLF recruitment to damage sites

**Figure 5:**
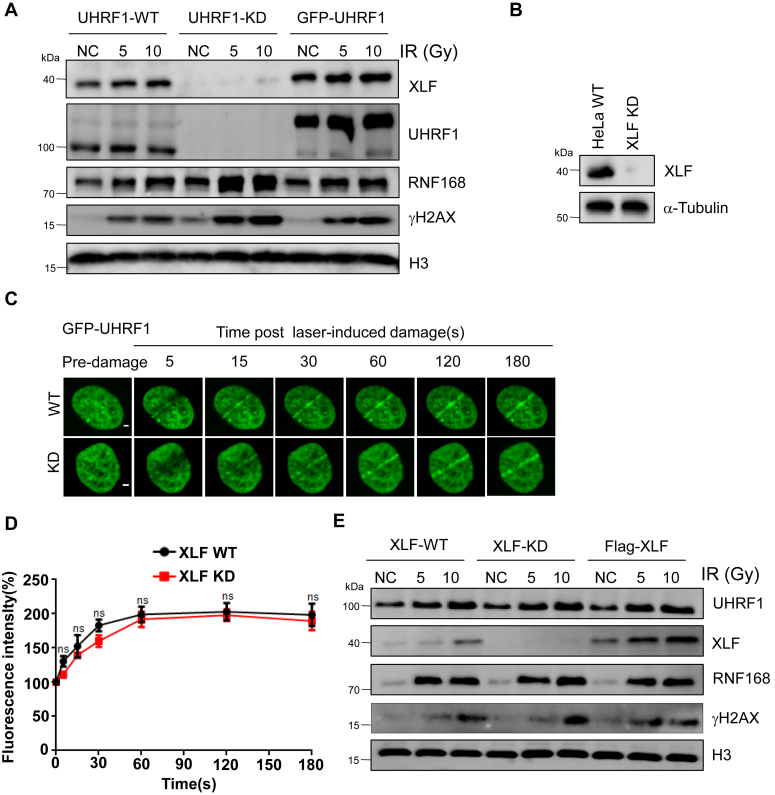
**UHRF1 promotes XLF recruitment to damage sites**. *A*, HeLa WT, UHRF1 knock down, and UHRF1 rescue cells were exposed to different doses of IR, and after a 30 min release, chromatin was extracted for Western Blot. *B*, XLF was knocked down in HeLa cells by CRISPR-Cas 9, and the XLF knockdown efficiency was determined by Western Blot. *C* and *D*, HeLa WT cells and XLF knock down cells were transfected with GFP-UHRF1. After 24 h, dynamic recruitment of UHRF1 to DNA damage tracks were monitored using laser micro-irradiation–coupled live-cell imaging, Scale bars represent 10 μm. *D*, we analyzed and quantified the percentage of fluorescence increase along 20 cellular DNA damage trajectories. *E*, HeLa WT, XLF knock down, and XLF rescue cells were exposed to different doses of IR, and after a 30 min release, chromatin was extracted for Western Blot.

Next, we extracted chromatin from WT and UHRF1 KD cells after irradiation and detected the recruitment of XLF to chromatin. We found that XLF was barely detected in the chromatin fraction in UHRF1 KD cells. And the recruitment of XLF to chromatin was restored by re-introduction of UHRF1 back into the cells (Fig. 5*A*). All these results proved that ubiquitination of XLF by UHRF1 promotes XLF localized to the DSBs sites to repair the broken DNA. To figure out whether function of UHRF1 depends on XLF, XLF was knocked down using Crispr-cas 9 technology followed by Western Blot to test the knockdown efficiency (Fig. 5*B*). Using laser micro-irradiation experiments, we confirmed that the loss of XLF does not affect the recruitment of UHRF1 to DSBs sites(Fig. 5, *C* and *D*). Chromatin extraction also showed that XLF did not affect the recruitment of UHRF1 to DNA damage site (Fig. 5*E*).

### UHRF1 inhibition sensitize cancer cells to DNA damage

**Figure 6:**
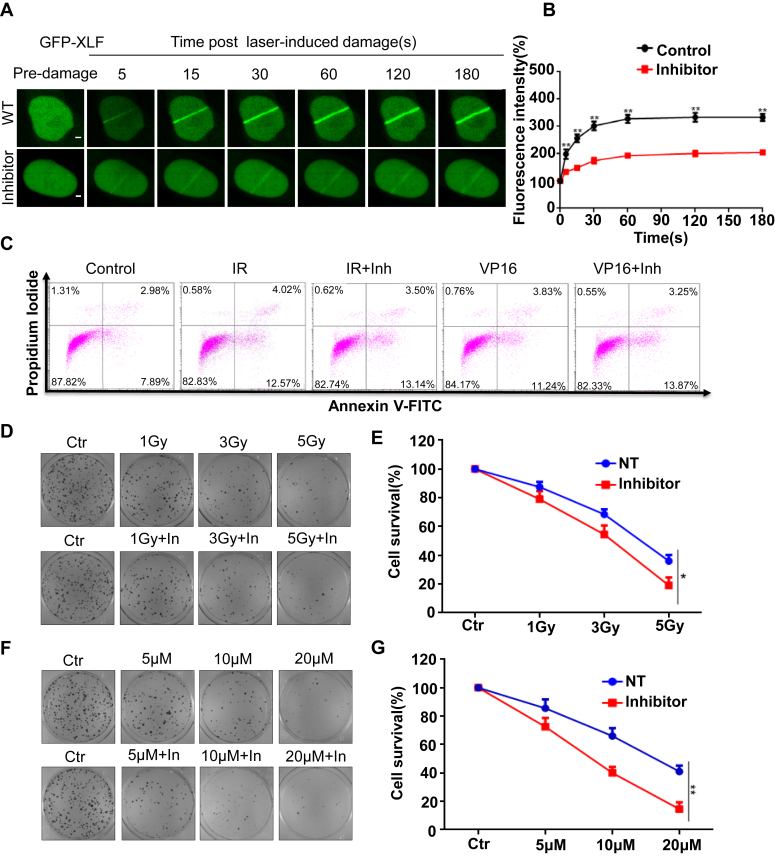
**UHRF1 inhibition sensitizes cancer cells to DNA damage**. *A* and *B*, HeLa WT cells were transfected with GFP-UHRF1, after 4 h of treatment with 20 μM NSC2320032003, dynamic recruitment of UHRF1 to DNA damage tracks were monitored using laser micro-irradiation–coupled live-cell imaging, Scale bars represent 10 μm. *B*, we analyzed and quantified the percentage of fluorescence increase along 20 cellular DNA damage trajectories. *C*, HeLa cells were irradiated with 5 Gy IR for 1 h, treated with 20 μM VP16 for 2 h, and then released for 2 h before being treated with 20 μM NSC232003 for 4 h. Apoptosis was assessed using the Annexin V-FITC apoptosis detection kit. *D* and *E*, HeLa cells were irradiated with IR for 6 h and then reseeded in 6-well plates. Twenty micromolars of NSC232003 was added, and cell clones were analyzed for relative viability by crystal violet staining. Representative images were captured using the Bio-Rad ChemiDoc XRS + system.∗*p* < 0.05. *F* and *G*, HeLa cells were treated with VP16 for 2 h and then released for 6 h before being reseeded in 6-well plates. Twenty micromolars of NSC232003 was added, and cell clones were analyzed for relative viability by crystal violet staining. Representative images were captured using the Bio-Rad ChemiDoc XRS + system.∗∗*p* < 0.01.

Previously, we demonstrated that UHRF1 participates in DNA damage repair by ubiquitinating XLF. Here, we utilized a highly efficient and cell-permeable UHRF1 inhibitor (NSC232003) (32). We found that the use of NSC232003 abolished the recruitment of XLF to DNA damage site (Fig. 6, *A* and *B*). Flow cytometry further confirmed that NSC232003 triggers apoptosis, and UHRF1 inhibition induces more cell apoptosis under DNA damage conditions (Figs. 6*C* and S4*A*). Colony formation assays showed that the addition of NSC232003 after IR and VP16 treatment further reduces the number of surviving cancer cells (Fig. 6, *D*–*G*). Overall, our findings indicate that UHRF1 ubiquitinates XLF *via* K63 linkage, facilitating the recruitment of XLF to DNA damage sites, which positively impacts cellular NHEJ repair (Fig. S5*A*).

## Discussion

The NHEJ pathway is one of the major error prone DNA repair pathways to promote human genomic rearrangements (33, 34). Core NHEJ factors XLF, PAXX, XRCC4, and LIG4 form a complex to execute the NHEJ repair progress (22, 35, 36). XLF KO mice were alive but showed radio-sensitivity and defects in lymphocytes (37). Interestingly, XLF and PAXX double KO mice were lethal, mimicking XRCC4- or Lig4-deficient mice, suggesting the redundant roles of these two factors in NHEJ (25). XLF also showed overlapping roles with several other factors such as ATM, DNA-PKcs, and MRI (23, 38, 39). Given the importance of the repair complex, how is it regulated to govern NHEJ remains of great interests to the field. Liu P *et al.* reported that Akt-mediated phosphorylation of XLF at T181 and impairs NHEJ (26). Here we reported that E3 ubiquitin ligase UHRF1 associated with XLF upon DNA damage (Fig. 2*E*) and mediated its K63, but not K48, linked ubiquitination (Fig. 3, *E* and *F*). Usually, polyubiquitin chains at different lysine residues were associated with different cellular functions, as K63-linked ubiquitination formed a binding hub to mediate protein–protein interactions or transmitted cellular signals, while K48-linked polyubiquitin chains were involved in proteasomal degradation. Although in some cases, K63 ubiquitylation could also trigger proteasome-mediated protein degradation (40). Interestingly, both K63- (3, 21) and K48- (41, 42) linked ubiquitination played crucial roles in the DNA damage response through different mechanisms. In our cases, polyubiquitination of XLF did not lead to protein degradation (Fig. 4*C*), instead promoted its recruitment to the DNA damage site (Fig. 4*E*). Further work could be done to identify the lysine residues on XLF and how these ubiquitin chains changed the conformation of the protein. Another interesting possibility is to find out whether ubiquitination of XLF enhances its binding with factors at DNA damage site such as other core NHEJ repair factors or even histones.

UHRF1 plays a vital role in maintaining the global genome methylation level through recruiting DNMT1 to replication forks to methylate the newly synthesized DNA (7, 43). UHRF1 KO mice were embryonic lethal (44). However, UHRF1 was also a proto-oncogene that overexpressed in several tumor tissues (43, 45, 46, 47) and high UHRF1 expression is a marker of poor prognosis in human KRAS mutant lung adenocarcinoma (48), while the exact role of UHRF1 in tumorigenesis remains elusive. Although UHRF1 was reported to have E3 ligase activity–independent roles to suppress AMPK activity and functions (49), its function in DSBs repair and maintaining genome stability largely depends on its enzymatic activity (21, 50). In our case, UHRF1 associated with XLF and mediated its polyubiquitination, however, whether the E3 ligase activity is required for the recruitment of XLF to DSBs site needs to be further investigated.

## Experimental procedures

### Cell culture

HeLa and HEK293T cells were cultured in Dulbecco’s modified Eagle’s medium (Gibco) supplemented with 10% fetal bovine serum (ExCell Bio) and 1% penicillin-streptomycin (OMACGENE). The humidified incubator is maintained at 37 °C with 5% CO2.

### Antibodies and reagents

The antibodies used in this study included the following: UHRF1(12387S, CST); XLF(A300-730A, BETHYL); RNF168(21393-1-AP, Proteintech); H3(ab1791, Abcam); GFP(M048-3, MBL); Flag(F1804, Sigma); Phospho-H2A.X (Ser139,80312S, CST); alpha Tubulin(sc-398103, Santa Cruz); BRCA1(9010S, CST); HA(M180-3, MBL); GAPDH(sc-32233, Santa Cruz); FK2(04-263, EMD Millipore); Anti-FLAG M2 agarose beads(B23102, Bimake). Other reagents such as etoposide, puromycin were purchased from Sigma-Aldrich; Chloroquine, NSC232003 were purchased from MedChemExpress; Cycloheximide, MG132 were purchased from Selleckchem.

### Plasmids and sgRNA

All plasmids and sgRNAs were transfected into cells using UltraFection 3.0 (FXP135-010, 4A Biotech) according to the manufacturer's instructions. The complementary DNA was used as a template to amplify UHRF1 and XLF through PCR, followed by cloning into p3 × FLAG-CMV10 or pEGFP-C1 vectors (Addgene). The HA-tagged ubiquitin plasmids and mutants were provided by Prof. Xingzhi Xu at Shenzhen University. The UHRF1 sgRNA sequence was constructed in the PX459/Puro vector (Addgene). The sgRNA sequences used are as follows:

UHRF1#1: 5′-CACCGGTGGATCCAGGTTCGGACCA-3′

UHRF1#2: 5′-CACCGGCCTGCAGAGGCTGTTCTAC-3′

XLF#1: 5′-CACCGTGAACAGGTGGACACTAGTG-3′

XLF#2: 5′-CACCGGTTGATGCAGCCATGGGCGT-3′

### Immunofluorescent staining

Cells were fixed with 4% paraformaldehyde, permeabilized with 0.5% Triton X-100, and, after cell blocking, incubated with primary antibodies (UHRF1 1:500; XLF 1:500) overnight at 4 °C. Cells were exposed to the appropriate Alexa Fluor 594-conjugated secondary antibodies (1:500) for 1 h at room temperature. After three washes with PBS containing 0.1% Tween 20, cell nuclei were stained with DAPI. Immunofluorescent images were captured using a Nikon confocal microscopy. All the gels were quantified by Image J software.

### Laser micro-irradiation

Cells were seeded in glass-bottom culture dishes and transfected with a GFP-tagged plasmid for 24 h. Subsequently, they were irradiated with a 365 nm pulsed nitrogen ultraviolet laser generated by a MicroPoint system (Andor) at a pulse rate of 16 Hz and 55% laser output. Delayed images were captured every 5 s over a duration of 180 s. The signal intensity along the irradiation path was quantified using ImageJ.

### Co-immunoprecipitation assay

For the whole cell lysate co-immunoprecipitation assay, after treatment, cells were harvested and incubated in NP-40 lysis buffer (20 mM Tris–HCl pH 8.0, 137 mM NaCl, 10% glycerol), supplemented with 1% NP-40 and protease inhibitor cocktail (Roche, Switzerland), on ice for 30 min. The mixtures were sonicated at 30% intensity (12 times, on ice, each for 5 s). Centrifuge the cell lysate at 4 °C 12,000 rpm for 15 min. The supernatants were collected and incubated with applicable beads at 4 °C for 10 to 12 h, then washed four times with ice-cold PBS. Finally, the samples were resuspended in 2×SDS loading buffer, denatured at 100 °C for 10 min, and stored at −20 °C.

### Colony formation assay

After treatment with IR or VP16 and subsequent release, cells were recounted and re-cultured in fresh medium for 2 weeks. The cells were then stained with crystal violet, and the number of colonies with >50 cells was counted.

### Apoptosis assay

The apoptosis assay was conducted using PI and Annexin-V-FITC staining kits (Beyotime, C1062M). After 20 min of incubation with PI and annexin-V FITC in the dark, the cells were resuspended in a binding buffer and examined using a flow cytometer.

### Denaturing lysis of cells for immunoprecipitation

After collecting cell samples, wash once with PBS. Then add denaturing cell lysis buffer (50 mM Tris (pH 7.5), 70 mM β-mercaptoethanol should be freshly added and the solution should be heated for 10 min after addition) and heat at 95 °C for 10 min. Add 4 times the volume of 1X cell lysis buffer (20 mM Tris (pH 7.5), 150 mM NaCl, 1 mM EDTA, 1 mM EGTA, 1% Triton X-100, 2.5 mM sodium pyrophosphate, 1 mM β-glycerophosphate, 1 mM Na3VO4, 1 μg/ml Leupeptin; 1 mM PMSF should be added before use) to the lysis buffer. Mix well and take 50 μl of the cell lysate, add 2×SDS loading buffer, and heat at 95 °C for 10 min to prepare the Input sample. Add pre-mixed Flag beads to the remaining lysate and incubate overnight on a rotating wheel at 4 °C. After incubation, centrifuge at 1000 rpm for 1 min at 4 °C. Discard the supernatant, wash the Flag beads five times with 500 μl of cell lysis buffer, and then resuspend in 2 × SDS loading buffer. Heat at 95 °C for 10 min to prepare the IP sample and store at −20 °C.

### *In vitro* ubiquitination assay

The recombinant His-UHRF1 and His-XLF was expressed in *Escherichia coli* and purified with a His-tag purification column (Beyotime). *In vitro* ubiquitination assays were performed with 2 ng E1 (UBE1) (UB-biotech), 10 ng E2 (UBE2N/Ubc13) (UB-biotech), 20 ng His-UHRF1, and 20 ng His-XLF in 40 μl of reaction buffer (50 mM Tris (pH 7.5), 2.5 mM MgCl2, 2 mM ATP, and 2 mM DTT). The reactions were carried out at 37 °C for 45 min and stopped by boiling in SDS sample buffer.

## Data availability

The data presented in this study are available on request from the corresponding author.

## Supporting information

This article contains supporting information.

## Conflicts of interest

The authors declare that they have no conflicts of interests with the contents of this article.
