# Supplementary material for: UHRF1-mediated ubiquitination of nonhomologous end joining factor XLF promotes DNA repair in human tumor cells
Source: J Biol Chem. 2024 Sep 27;300(11):107823. doi: 10.1016/j.jbc.2024.107823 (PMC11530599; doi:10.1016/j.jbc.2024.107823)
Supplement: Supplementary Figure [file mmc1.pptx]

## Slide 1
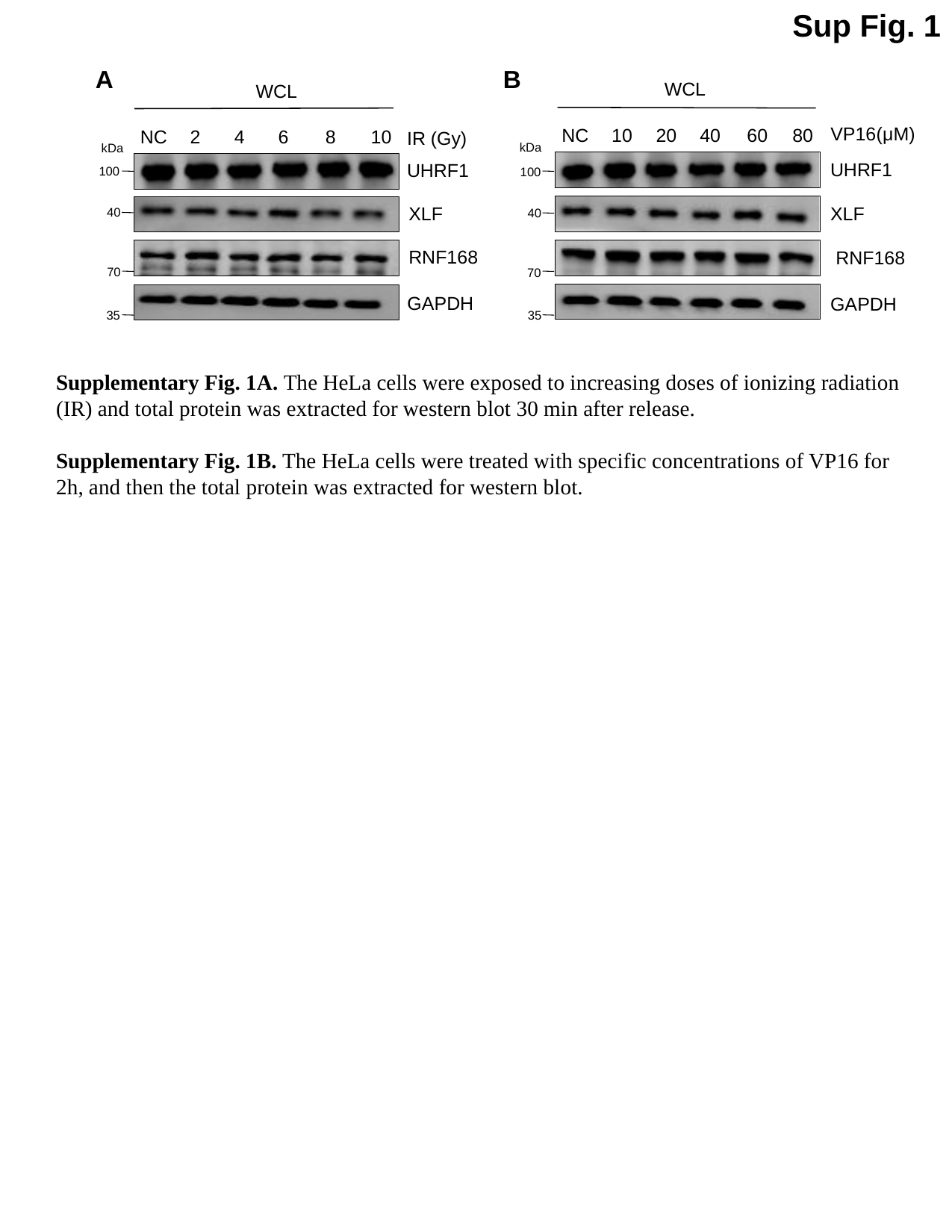

Sup Fig. 1
A
B
WCL
WCL
VP16(μM)
| NC | 10 | 20 | 40 | 60 | 80 |
| --- | --- | --- | --- | --- | --- |
| NC | 2 | 4 | 6 | 8 | 10 |
| --- | --- | --- | --- | --- | --- |
IR (Gy)
kDa
kDa
UHRF1
UHRF1
 100
 100
XLF
XLF
40
40
RNF168
 RNF168
70
70
GAPDH
GAPDH
35
35
Supplementary Fig. 1A. The HeLa cells were exposed to increasing doses of ionizing radiation (IR) and total protein was extracted for western blot 30 min after release.
Supplementary Fig. 1B. The HeLa cells were treated with specific concentrations of VP16 for 2h, and then the total protein was extracted for western blot.

## Slide 2
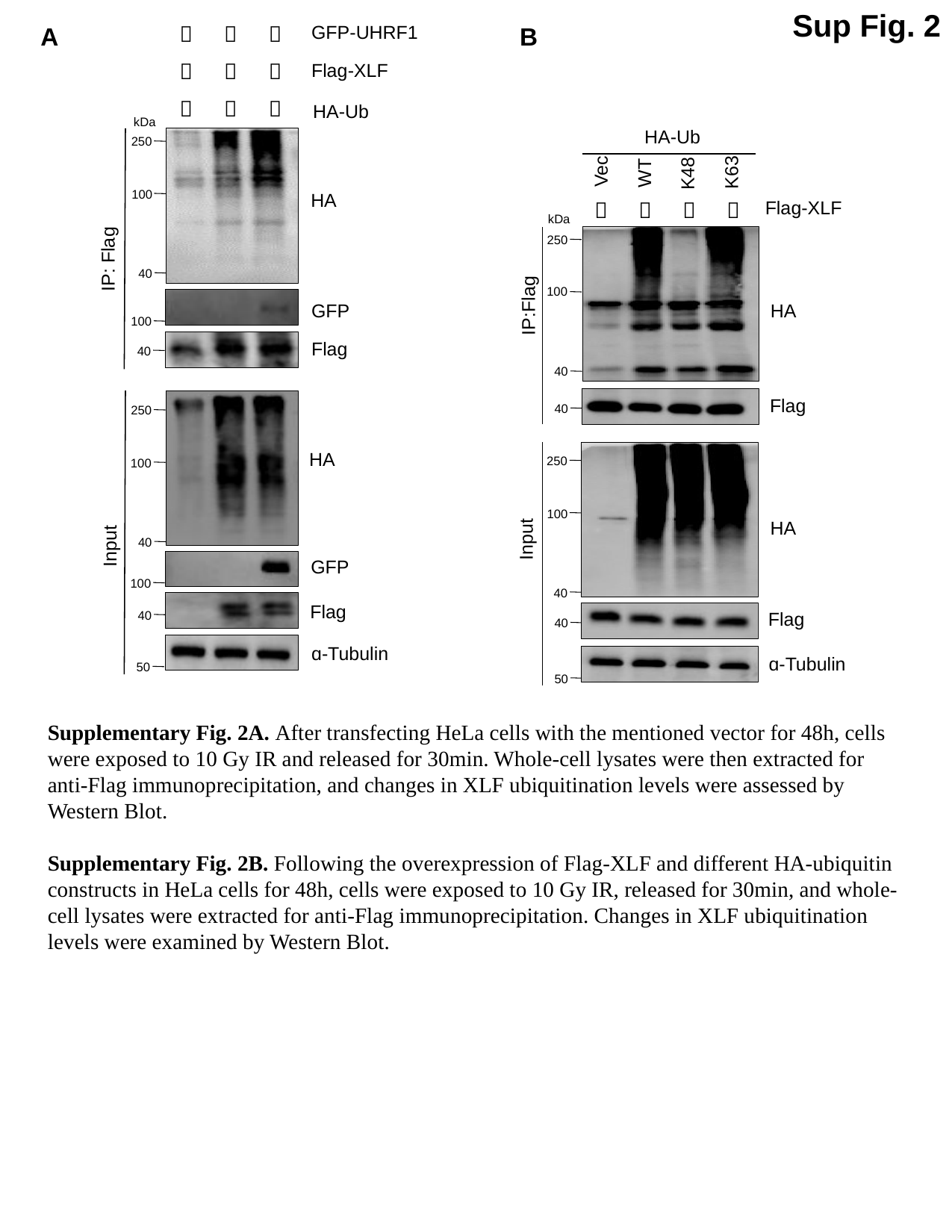

Sup Fig. 2
| － | － | ＋ |
| --- | --- | --- |
| － | ＋ | ＋ |
| － | ＋ | ＋ |
A
GFP-UHRF1
B
Flag-XLF
HA-Ub
kDa
HA-Ub
 250
K63
WT
K48
Vec
100
HA
| ＋ | ＋ | ＋ | ＋ |
| --- | --- | --- | --- |
Flag-XLF
kDa
 250
IP: Flag
40
100
HA
GFP
 IP:Flag
 100
Flag
40
40
Flag
40
 250
HA
 250
100
100
HA
Input
 Input
40
GFP
 100
40
Flag
40
Flag
40
ɑ-Tubulin
ɑ-Tubulin
50
50
Supplementary Fig. 2A. After transfecting HeLa cells with the mentioned vector for 48h, cells were exposed to 10 Gy IR and released for 30min. Whole-cell lysates were then extracted for anti-Flag immunoprecipitation, and changes in XLF ubiquitination levels were assessed by Western Blot.
Supplementary Fig. 2B. Following the overexpression of Flag-XLF and different HA-ubiquitin constructs in HeLa cells for 48h, cells were exposed to 10 Gy IR, released for 30min, and whole-cell lysates were extracted for anti-Flag immunoprecipitation. Changes in XLF ubiquitination levels were examined by Western Blot.

## Slide 3
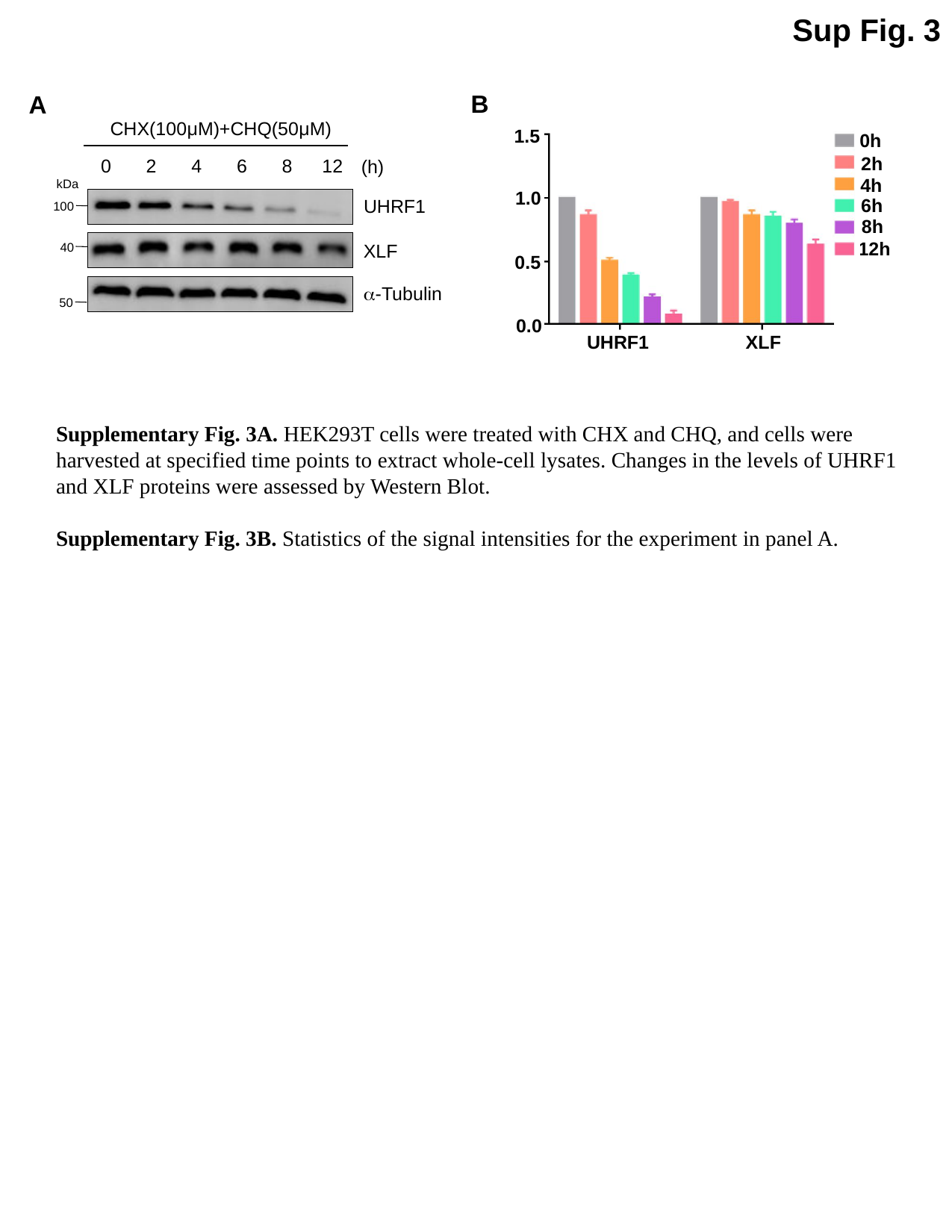

Sup Fig. 3
B
A
CHX(100μM)+CHQ(50μM)
1.5
0h
2h
4h
6h
8h
12h
1.0
0.5
0.0
UHRF1
XLF
(h)
| 0 | 2 | 4 | 6 | 8 | 12 |
| --- | --- | --- | --- | --- | --- |
kDa
UHRF1
 100
 40
XLF
a-Tubulin
 50
Supplementary Fig. 3A. HEK293T cells were treated with CHX and CHQ, and cells were harvested at specified time points to extract whole-cell lysates. Changes in the levels of UHRF1 and XLF proteins were assessed by Western Blot.
Supplementary Fig. 3B. Statistics of the signal intensities for the experiment in panel A.

## Slide 4
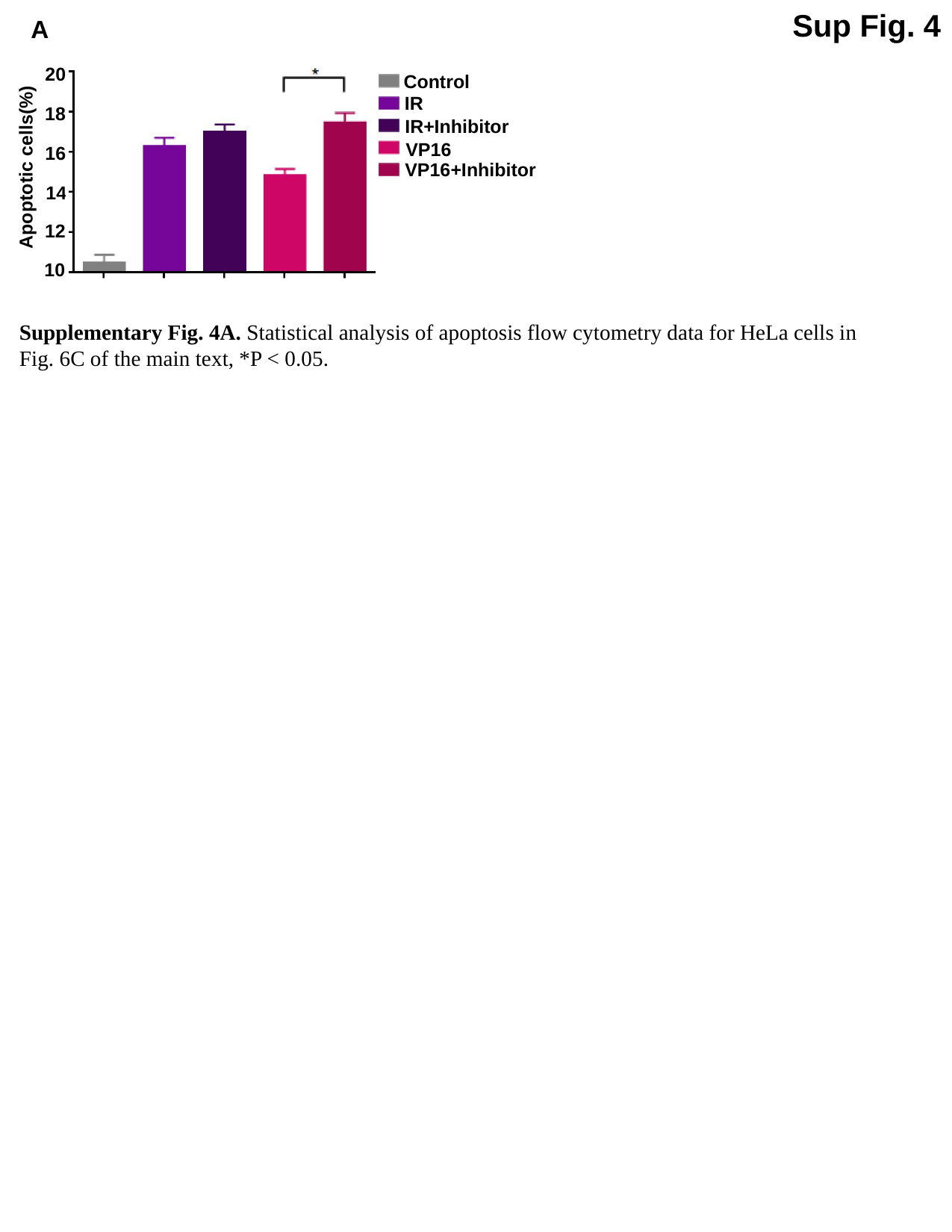

Sup Fig. 4
A
20
Control
IR
IR+Inhibitor
VP16
VP16+Inhibitor
18
16
Apoptotic cells(%)
14
12
10
Supplementary Fig. 4A. Statistical analysis of apoptosis flow cytometry data for HeLa cells in Fig. 6C of the main text, *P < 0.05.

## Slide 5
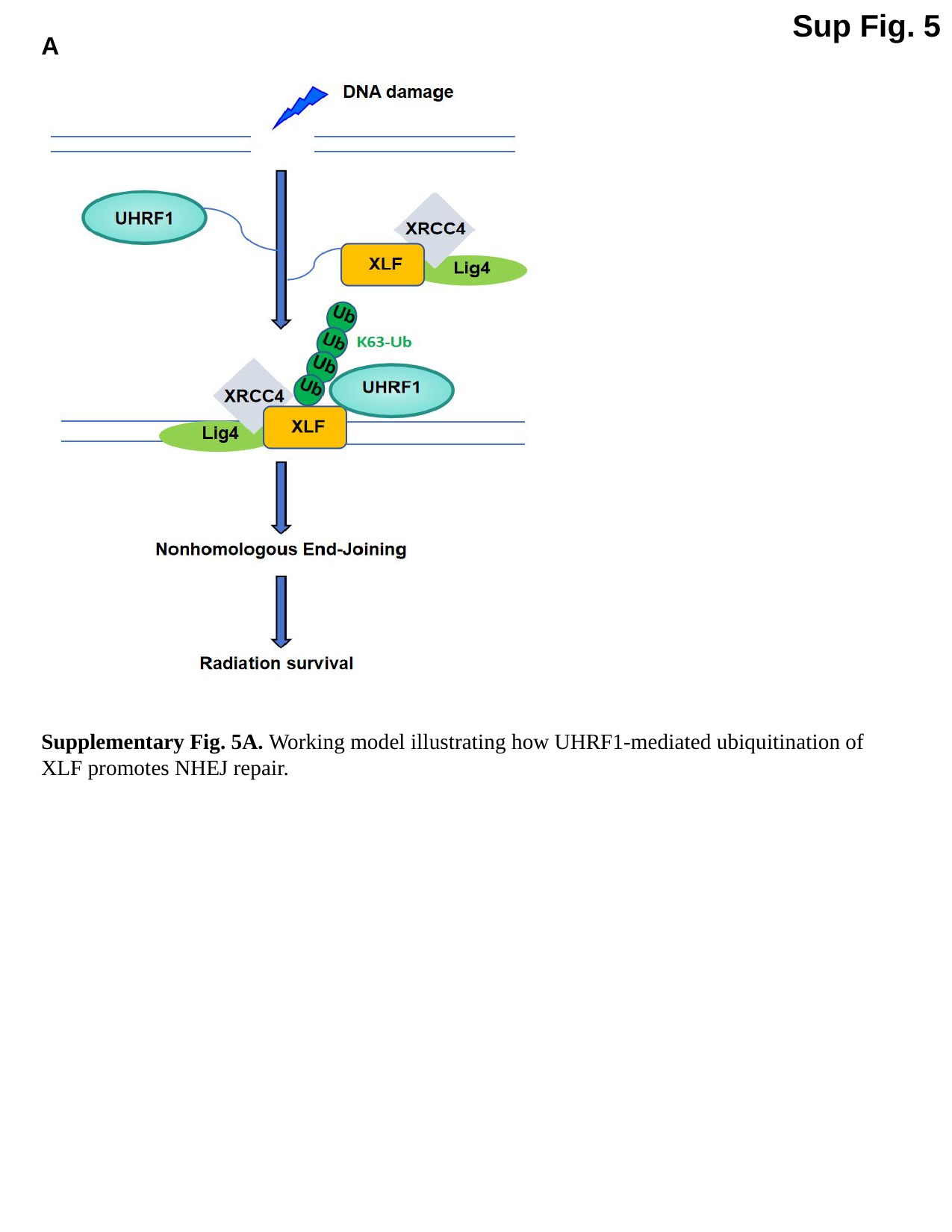

Sup Fig. 5
A
Supplementary Fig. 5A. Working model illustrating how UHRF1-mediated ubiquitination of XLF promotes NHEJ repair.
